# Information in a Network of Neuronal Cells: Effect of Cell Density and Short-Term Depression

**DOI:** 10.1155/2016/2769698

**Published:** 2016-06-14

**Authors:** Valentina Onesto, Carlo Cosentino, Enzo Di Fabrizio, Mario Cesarelli, Francesco Amato, Francesco Gentile

**Affiliations:** ^1^Department of Experimental and Clinical Medicine, University of Magna Graecia, 88100 Catanzaro, Italy; ^2^King Abdullah University of Science and Technology, Thuwal 23955-6900, Saudi Arabia; ^3^Department of Electrical Engineering and Information Technology, University of Naples, 80125 Naples, Italy

## Abstract

Neurons are specialized, electrically excitable cells which use electrical to chemical signals to transmit and elaborate information. Understanding how the cooperation of a great many of neurons in a grid may modify and perhaps improve the information quality, in contrast to few neurons in isolation, is critical for the rational design of cell-materials interfaces for applications in regenerative medicine, tissue engineering, and personalized lab-on-a-chips. In the present paper, we couple an integrate-and-fire model with information theory variables to analyse the extent of information in a network of nerve cells. We provide an estimate of the information in the network in bits as a function of cell density and short-term depression time. In the model, neurons are connected through a Delaunay triangulation of not-intersecting edges; in doing so, the number of connecting synapses per neuron is approximately constant to reproduce the early time of network development in planar neural cell cultures. In simulations where the number of nodes is varied, we observe an optimal value of cell density for which information in the grid is maximized. In simulations in which the posttransmission latency time is varied, we observe that information increases as the latency time decreases and, for specific configurations of the grid, it is largely enhanced in a resonance effect.

## 1. Introduction

Networks of nerve cells are complex systems in which a large number of components combine to yield collective phenomena with improved abilities in contrast to simple components of that system [1–6]. The human brain itself is a grid or a network of bewildering complexity where 10^12^ neurons cluster in three-dimensional architectures. The unprecedented functions of human brain, including self-consciousness, language, and the development of memory, may depend less on the specialization of individual neurons and more on the fact that a large number of them interact in a complex network [2, 3, 5, 6]. The human brain and mechanisms of information propagation through neural nets are being heavily investigated in the last years. Emerging nanotechnologies, whereby surfaces with a controlled nanotopography can regulate and guide the organization of neuronal cells into complex networks [7–13], advancements in traditional disciplines, that is, computer science and information theory [6, 14–19], and the combination of the two [5], may provide scientists with new tools to elucidate the mechanisms through which the brain marshals its millions of individual nerve cells to produce behavior and how these cells are influenced by the environment.

The exchange of information between individual neurons is mediated by a cascade of chemical to electrical signals which travel across the gap (synaptic cleft, approximately 20 nm wide) between those neurons [20]. At similar synapses, an action potential generated near the cell body propagates down the axon where it opens voltage-operated Ca^2+^ channels. Ca^2+^ ions entering nerve terminals trigger the rapid release of vesicles containing neurotransmitter, which is ultimately detected by receptors on the postsynaptic cell [20]. The described process continues repeatedly until the response at the postsynaptic sites reaches and surpasses a limiting value (i.e., a threshold); then, the target neuron produces an impulse (an action potential) that propagates in turn to another neuron. Noticeably, information is encoded by the frequency of the action potentials generated by the neurons rather than by their intensity [21]. Individual neurons and electrical activity thereof are correctly described by the celebrated leaky integrate-and-fire model in which the membrane potential of a neuron *ϕ* obeys a function of the sole time [21–25]:

(1) $$\begin{array}{c} C_{m}\frac{d\phi \left(t\right)}{dt}=-g_{l}\left(\;\phi \left(\;t\;\right)-\varphi \;\right)+I_{stim}\left(\;t\;\right), \end{array}$$

where *C*_*m*_ is the capacitance of *l*, the membrane, *g*_*l*_ is its conductance, and *φ* is the resting potential of the neuron. In (1), the current *I*_stim_(*t*) represents the stimulus that excites the neuron until the membrane potential reaches a threshold *θ*; then, an action potential AP is generated and the system is maintained for a refractory time at rest, in which (1) does not hold anymore, and this accounts for the short-term synaptic depression of the neuron [22]. Notice that one can multiply both terms of (1) by the reciprocal of the conductance *R* = 1/*g*_*l*_, which yields a different form of (1):

(2) $$\begin{array}{c} \tau \frac{d\phi \left(t\right)}{dt}=-g_{l}\left(\;\phi \left(\;t\;\right)-\varphi \;\right)+RI_{stim}\left(\;t\;\right) \end{array}$$

in which *τ* = *RC*_*m*_ is the time constant in a circuit theory interpretation of the neuron [21]. Equation (2) is used to predict the time evolution of individual neurons. Similarly, neurons in a grid are described by a set of coupled differential equations that generalize the model above to an ensemble of a large number of simple units connecting to each other. In contrast, in neural mass models, the activity of the entire neural population is lumped in a limited and generally low number of variables or parameters, in a statistical approach [21, 26]. These parameters are related to the moments of the distributions that are used to describe the neural population and may be sometimes coincident with the sole center of mass. While advantageous for mathematical convenience and computational tractability, neural mass models and their more sophisticated evolutions that have been developed over time (including mean field models and neural field models) are however based on an approximation and may therefore fail to resolve the dynamics of a system of neurons over each of its scales.

Here, we revise the integrate-and-fire model in the version proposed by De La Rocha and Parga [22] to extend the analysis to a bidimensional set of neurons in a grid. We use information theory variables, including the Shannon information entropy, described, for example, in [14, 16, 27, 28] and recapitulated in the following part of the paper, to determine the response of the network associated with an external stimulus. The intensity and distribution of information over the network are determined as a function of the total number *𝒩* of neurons in the grid (thus, cell density) and time of synapse recovery after the stimulus (thus, short-term depression). In what follows, neurons are connected through not-intersecting edges; in doing so, the degree of the graph would not depend on the number of nodes in the graph. Moreover, the maximum intermodal distance is upper bounded and maintained below a cut-off distance which represents an ideal synaptic length, whereby all connections greater than the prescribed cut-off are disrupted. In simulations where the number of nodes is varied over a significant range, we observe two different regimens of information dynamics in the grid: in the low cell density range, the information quality and density in a grid increase with *𝒩*; in the high cell density range, the information content in the grid increases less rapidly than the total number of cells, meaning that the information quality and density decrease with *𝒩*. For intermediate cell densities (i.e., when all connections in the graph are realized) the information density and quality in the grid reach a maximum. In simulations in which the posttransmission latency time is varied, we observe that information increases as the latency time decreases. More important than this, we observe that when the average firing rate of individual neurons (i.e., a property of single neurons in isolation) is an integer number of times greater than the characteristic signalling frequency in the grid (i.e., a property of a set of neurons in cooperation), the transport of information is largely enhanced, similarly in concept to the resonance of a mechanical system. These data reinforce the view that the organization of neural cells in a network and the topology of the network itself play a major role in the spread of information in a complex of those cells.

## 2. Methods

### 2.1. Generating Networks of Neurons in the Plane

**Figure 1:**
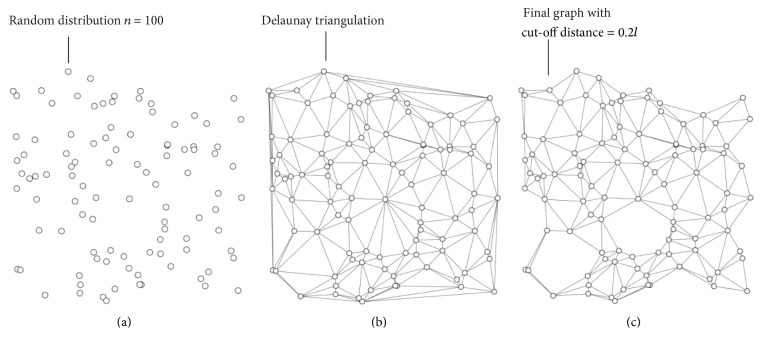
A random distribution of nodes in a plane (a); nodes are connected through a Delaunay triangulation, that guarantees that the number of edges varies linearly with the number of nodes (b); the resulting graph upon after removal of the internodal distances smaller than a cut-off distance, which is here 0.2 times the length of the lattice.

We consider *𝒩* neural cells uniformly distributed in a square domain with edge *l* (Figure 1). Individual nodes are indicated with the symbol *𝒫*_*i*_:  *i* = 1,…, *𝒩*. In what follows, we may use interchangeably the terms neural cells, neurons, and nodes. Nodes are connected through not-intersecting lines or edges, which are the vertices of the Delaunay triangulation of those nodes in the plane (Figure 1). The number of edges *υ* resulting from a similar triangulation varies linearly with *𝒩*, with *υ* = 3*𝒩* − 3 − *k*, where *k* is the number of points on the convex hull of the original data set *𝒫*. Thus, the degree of the graph *𝒟* would not depend on the absolute number of cells and for sufficiently large *𝒩*, *𝒟* ~ 6. The Delaunay triangulation is the dual graph of the Voronoi diagram. From this, certain properties arise, including the following: (i) the Delaunay triangulation of *𝒫* maximizes the minimum angle over all triangulations of *𝒫*; (ii) the circumcircle of any triangle in the Delaunay triangulation is empty (contains no sites of *𝒫*); (iii) the closest pair of sites in *𝒫* are neighbors in the Delaunay triangulation; (iv) the minimum spanning tree of *𝒫* is a subgraph of the Delaunay triangulation of *𝒫*. Recalling that the* Euclidean minimum spanning tree* is the graph with minimum summed edge length that connects all points in *𝒫*, the Delaunay triangulation of a set of neurons has therefore some biological sense. A system of neural cells on a substrate is likely to develop synapses following the shortest path between those cells, thus maintaining the energy of the ensemble at a minimum, which is equivalent to the conditions from (i) to (iv) above and especially to property (iv). For each element (node) *i* in *𝒫*, we define a bundle of *𝒫*_*i*_ and indicate with *ℬ*_*i*_ the subset of nodes in *𝒫* that are connected to *𝒫*_*i*_. The information about the connections amid the nodes in a graph is contained in the adjacency matrix *A* = *a*_*ij*_, where the indices *i* and *j* run through the number of nodes *𝒩* in the graph. *a*_*ij*_ = *d*_*ij*_ (where *d*_*ij*_ is the Euclidian distance function) if there exists a connection between *i* and *j*; *a*_*ij*_ = *∞* otherwise. In the analysis, reciprocity between nodes is assumed, and thus if information can flow from *i* to *j*; it can reversely flow from *j* to *i*. In the framework of graph theory, we call a similar network an undirected graph. Notice that this property translates into symmetry of *A* with *a*_*ij*_ = *a*_*ji*_. Moreover, *a*_*ii*_ = *∞*.

### 2.2. Neural Signalling

**Figure 2:**
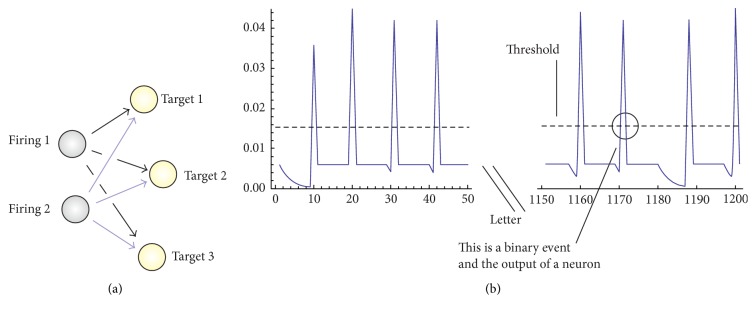
Nodes are divided into firing, delivering a signal, and target, receiving a signal: a target neuron can receive current pulses from multiple sources; in turn a firing neuron can deliver current pulses to multiple targets (a); the sum of multiple stimuli in a neuron modifies the potential across its membrane until it surpasses a limiting or threshold value: in this circumstance, the neuron generates an action potential (b).

Each node in the network sends and receives information and this process is mediated through the integrate-and-fire model and (2). In the Equation, *I*_stim_(*t*) is the stimulus associated with a specific neuron, which is the target. Assuming linearity, *I*_stim_(*t*) is given by the superposition of current pulses *J* generated by all the neurons in a bundle that fire on target neuron, which we shall herein designate as *j*, (Figure 2(a)) and thus

(3) $$\begin{array}{c} I_{stim}\left(\;t\;\right)=\overset{B}{\underset{i}{\sum }}\zeta \left(\;d_{ij}\;\right)J_{i}\overset{rel}{\underset{k}{\sum }}\delta \left(\;t-t_{i}^{k}\;\right), \end{array}$$

where rel is the number of neurotransmitter release events (i.e., the total number of spikes in a train), *δ* is the Dirac delta function, and *t*_*i*_^*k*^ is the timing of individual pulses. In (3), *ζ* is a damping term which accounts for the internodal distance *d*_*ij*_ and the arrival time delay from *i* to *j*. The pulses that repeatedly excite a target neuron amplify the membrane potential in that neuron until it exceeds a threshold *θ* and an action potential is generated (Figure 2(b)). A closed form solution of (2) along with condition (3) exists with

(4) $$\begin{array}{c} \phi \left(\;t\;\right)=\varphi +\overset{B}{\underset{i}{\sum }}\zeta \left(\;d_{ij}\;\right)J_{i}e^{-\left(t-t_{i}^{k}\right)/\tau }\overset{rel}{\underset{k}{\sum }}H\left(\;t-t_{i}^{k}\;\right), \end{array}$$

where *H* is the Heaviside function and (4) applies in the subthreshold regime, that is, for *ϕ* < *θ*. When *ϕ* = *θ*, the target is activated (in what resembles a binary event) and the resulting action potential is converted into an impulse *J* that propagates in turn from the target neuron (now, the firing) to all those nodes that are connected to it in cascade. Upon the discharge of the action potential, the neuron is maintained at rest for a refractory or resting time *τ*_*r*_, which reproduces the short-term depression and in which any activity is inhibited. The sequence of this 3-step fire/receive/fire process, in which each neuron alternates from being a firing to a target neuron, reiterates until either all nodes are covered and the passage of the signal over the network is completed, or the intensity of the signal becomes vanishingly small. The balance/unbalance between the time constant of each neuron and the refractory time and the number of neurons in the grid is reflected by the timing of spikes that may be recorded on the individual sites and on the entire grid.

### 2.3. Encoding Information in the Network

**Figure 3:**
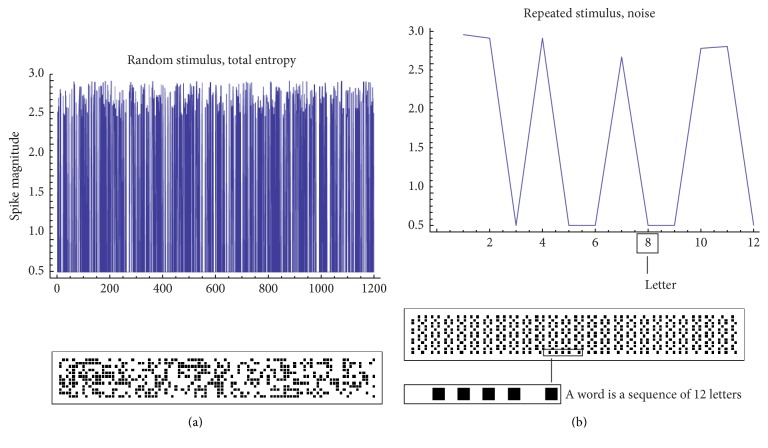
The entire grid is stimulated with a signal that can be random (a) or periodic (b). From a random long sample of stimuli, one may derive the total entropy of the spike train. Similarly, the noise entropy is the variability of the spike train in response to the sample of repeated stimuli. Information is as the difference between the total and the noise entropy.

**Figure 4:**
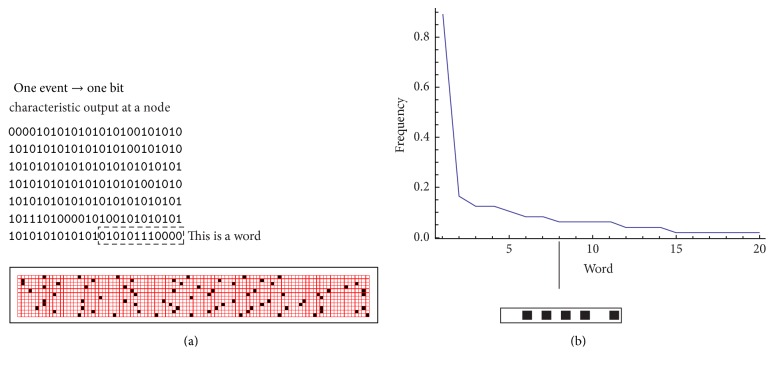
Entropy is a measure of the variability of a signal. In each neural site, the signal is registered like a sequence of 0/1 binary events: signals are encoded in bits (a). The number of occurrences of a bit in a sequence is the probability that a bit is generated, from the probability; entropy may be derived (b).

The temporal sequence of pulses or spikes which propagates along the grid encodes the information transmitted over that grid, which can be represented through the sole Shannon information entropy [17]. The goal of this section is to give a model independent estimate of entropy and information in neural spike trains as they encode dynamic signals. The spike train of individual neurons in response to a (sufficiently) long sample of stimuli may vary. A similar variability is described by the* total* entropy of the spike train, that is, *S* ^*o*^(Δ*τ*), where Δ*τ* is the time bin in which one chooses to discretize the spike train (Figure 3(a)). Similarly, the conditional or* noise* entropy *S* ^*N*^(Δ*τ*) is the variability of the spike train in response to a sample of repeated stimuli, in which the entire sequence of the input signal is given by the repetition of the same pattern (Figure 3(b)). The information that the spike train provides about the input is the difference between these entropies, *I* = *S* ^*o*^ − *S* ^*N*^. Δ*τ*/*τ* is a measure of the number of times that a neuron generates a burst in Δ*τ* (i.e., the number of spikes); in these aspects, Δ*τ* is the* resolution* with which a signal is transmitted or received. If the time bin Δ*τ* is sufficiently small (comparable to the time constant of a neuron, Δ*τ* ~ *τ*), then it may take discrete 0/1 values. In this limit, Δ*τ* is a binary variable and the entropy and information thereof may be expressed in units of bits. Consider a temporal window *T* in which the signal is transmitted. This segment is a word which contains *T*/Δ*τ* symbols or letters. The transmission of the entire signal is completed in a number of *n* repetitions of *T*. The total entropy is derived all over the words *w* in which the signal is segmented, and thus

(5) $$\begin{array}{c} S^{o}\left(\;\Delta \tau \;\right)=-\underset{w}{\sum }p\left(\;w\;\right){log}_{2}p\left(\;w\;\right), \end{array}$$

where *p*(*w*) represents the frequency of occurrences of *w* over all the runs *T* (Figure 4). In contrast, the noise entropy is averaged over *T*, and thus

(6) $$\begin{array}{c} S^{o}\left(\;\Delta \tau \;\right)={\;\langle \;-\sum_{w}p\left(\;w\;\right){log}_{2}p\left(\;w\;\right)\;\;\rangle }_{T}. \end{array}$$

**Figure 5:**
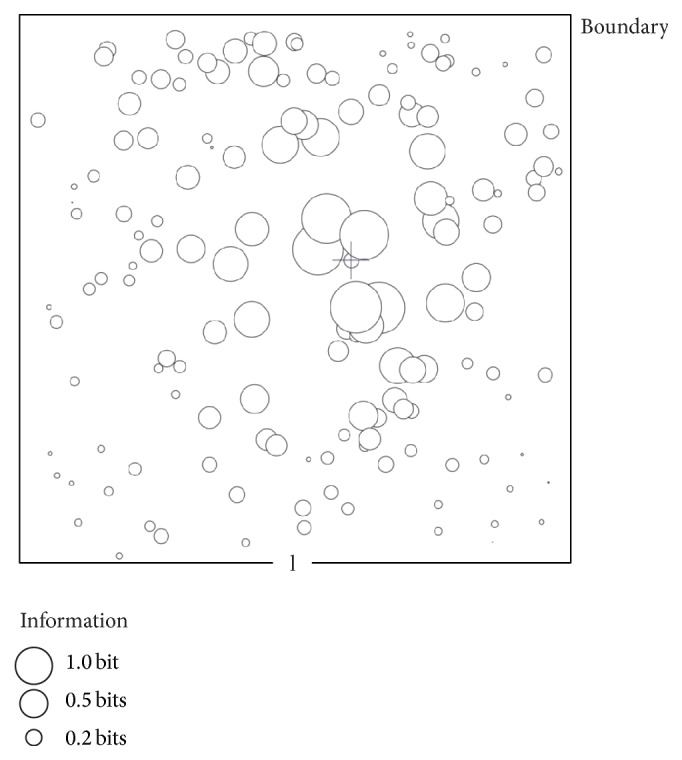
Information is derived in each node of the network. Here, we present information as circles or spheres in which the diameter of the sphere is proportional to the total information conveyed through a node over time.

Thus, the information carried by the stimulus is a difference between entropies. To operate a similar estimate in practice, we (i) generate a random sample of impulses (as in Figure 3(a)) that stimulates a neuron in the grid and in cascade all neurons in the grid and this is described by the integrate-and-fire model and Section 2.2 (the length of the stimulus is *nT*). We (ii) derive the response to the stimulus in all the active sites of the network using (1) to (4); then, we (iii) determine the entropy of the response *S* ^*o*^ in those sites using (5) and (6) (to do so, we determine the frequency, i.e., the probability *p*(*w*) of a word calculated over all the words in the response as in Figure 4). We (iv) repeat the same procedure where the signal is now a time-locked, periodic repetition of the same random set which varies over *T* (as in Figure 3(b)), repeated over *n* cycles, to obtain *S* ^*N*^. Direct calculation (v) yields the information *I* transported over all the nodes of the grid (as in Figure 5, i.e., further described in Section 3 below and comments thereof).

## 3. Results

### 3.1. The Information Transported over the Grid: The Spatial Dependency

We consider a grid composed by *𝒩* neurons where *𝒩* is varied in the 25–200 range and the topology of the grid is described in Section 2. Initially, the entire system is placed in the initial condition, where the signal and information are zero everywhere in the grid. These conditions are then perturbed with a random uncorrelated stimulus that is applied to a node *o* randomly chosen among those comprised in a small neighborhood of the center of network. In all cases, the initial disturbance propagates outward from the initial position to the boundaries of the system. This stimulus serves to derive the total entropy of the signal transmitted over the grid. Then, the system is perturbed with a periodic correlated stimulus, from which the noise entropy is derived. The difference between the total entropy and the noise yields an estimate of the information transmitted all over the nodes of the network. In the simulations, we posit with [22] that the neural time constant is *τ* = 3 ms, the membrane capacitance *C*_*m*_ = 300 pF, the amplitude of a pulse *J*/*C*_*m*_ = 0.25 mV, and the resting and threshold potentials, *φ* = 6 mV and *θ* = 9 mV, respectively; moreover, the time bin Δ*τ* = 3 ms, the length of a word *T*/Δ*τ* = 12, the number of trials *n* = 400, and the length of the grid *l* = 200 *μ*m. For the present case, the refractory time is maintained fixed as *τ*_*r*_ = 10*τ*. The definition and significance of the symbols above are provided throughout the paper and in a separate list of symbols.

**Figure 6:**
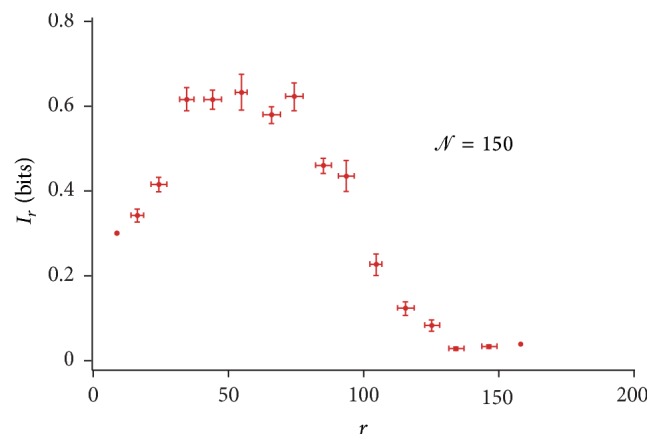
Information delivered through individual nodes can be integrated over a circumference of radius *r*, where the radius is the distance from the initial point of transmission. The integral is a measure of information transported at a specific length from the center of the network. In the diagram, you may observe three different regimens of information transmission. In the intermediate regimen, the total information conveyed through the grid is constant.

**Figure 7:**
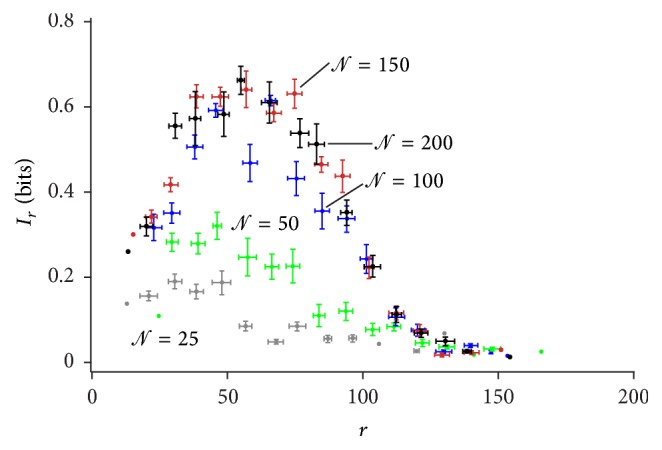
The information transported at a distance *r* from the center of the grid is represented as a function of the number *𝒩* of nodes in the grid. For small *𝒩*, information increases with *𝒩*. For large *𝒩*, saturation in the grid hampers information growth.

Figure 5 reproduces the information transmitted over the network for a specific configuration (here, *𝒩* = 100), where the circles at any node have a diameter that is proportional to the information transferred through that node in bits. Notice that the modulus of information is high in close proximity to the source of stimulation; then, it smoothly decays moving from the center to the periphery of the grid. The total information *I*_*r*_ transmitted at a specific distance *r* from the center is displayed in Figure 6 for a number of nodes in the grid *𝒩* = 100, considered as an example. In the diagram, the error bars are determined over at least 20 simulations per data point. We observe three regimens of transmission in the grid: (i) for small *r*, the information varies linearly with the distance; (ii) for intermediate *r*, *I*_*r*_ displays a constant value, meaning that the information carried by the stimulus would not depend on the distance from the stimulus; (iii) for large *r*, *I*_*r*_ decreases with 1/*r*. The described regimens result from the competition between the degradation of information and the number of nodes encountered at a specific *r* over which *I*_*r*_ is determined. In the linear regime (i), the number of nodes increases more rapidly than the degradation of information at a specific *r*, and thus we can register an overall growth of *I*_*r*_ with *r*. The remaining regimens may be explained similarly. The information *I*_*r*_ against the radius *r* is shown for different numbers *𝒩* of neurons in the network in Figure 7. From this, we can make two observations. The first is that the leading edge of the distribution moves to increasing values of *r* for increasing *𝒩*. The second is that the integral of the distribution is higher for large values of *𝒩*. And thus the center of mass of information moves from the center to the periphery of the domain with *𝒩*, but this effect is progressively reduced with the number of neurons in the grid. The effect of *𝒩* on *I* is even more visible on reporting the information integrated over the entire grid against *𝒩*, and this is described in the following section.

### 3.2. The Information Transported over the Grid: Effect of the Number of Neurons *𝒩*

**Figure 8:**
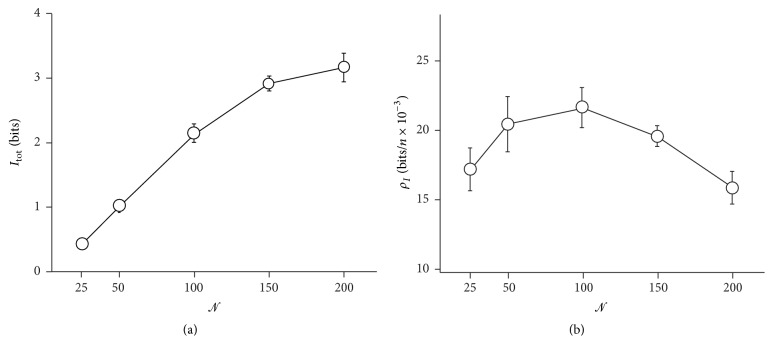
The information integrated over the entire grid as a function of number of neurons *𝒩* in the grid. Information increases linearly with *𝒩* for small *𝒩*; differently, information growth is hindered for large *𝒩*: for the present configuration *𝒩* = 100 marks the transition from small to large (a). The total information can be divided per the number of neurons and this yields the information density in the grid; on reporting the information density as a function of *𝒩*, one may observe that an optimal number of nodes exists at which it reaches a maximum, here, *𝒩* = 100 (b).

The overall information *I*_tot_ transported through the grid is reported in Figure 8(a) as a function of *𝒩*. You may notice that *I*_tot_ increases linearly with *𝒩* for small *𝒩*; differently, for sufficiently large *𝒩*, the propagation of information is retarded and *I*_tot_ grows less rapidly than *𝒩*. On dividing *I*_tot_ per the number of nodes, one obtains the information density *ρ*_*I*_ in the grid; that is an indication of how efficiently a message travels through the network and this is reported in Figure 8(b). From Figure 8(b) we observe that that information per node increases to reach a maximum at *𝒩* = 100; then, it decreases meaning that even if the information content as a whole continues to rise, the information density and quality progressively diminish. This is easily explained considering the number of active connections that exist among the nodes of the grid. If we have *𝒩* neurons uniformly distributed over a surface, the maximum internodal distance in a Delaunay cell of those neurons shall be on average

(7) $$\begin{array}{c} \delta_{n}=\sqrt{2}\frac{l}{\sqrt{N}-1}. \end{array}$$

**Figure 9:**
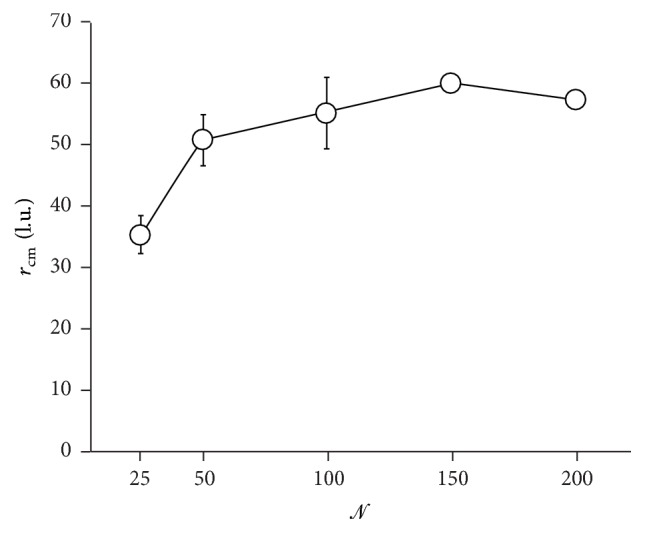
The position of the center of mass of information in the network as a function of number of neurons. It represent the distance at which information is transmitted. Depending on the effective links among neurons in a grid, there exists an optimal value of *𝒩* for which information in the domain travels the maximum distance.

For *𝒩* < 100, nodes in the grid are sparse and they are separated by a distance *δ*_*n*_ that is on average larger than the cut-off distance, *δ*_*n*_ > *δ*_co_, and thus fewer neurons will develop connecting synapses. The number of internodal connections is a monotonic, nondecreasing function of *𝒩* (7) and this would explain the behavior of *ρ*_*I*_ for *𝒩* < 100. Differently, for *𝒩* = 100, neurons in the grid are sufficiently dense and the majority of internodal connections are preserved (*δ*_*n*_ < *δ*_co_). For any *𝒩* larger than 100, the number of connections per neuron increases slowly with *𝒩* and approaches the theoretical value *𝒟* = 6. In this range, we observe a degradation of information quality with *𝒩*. The results here presented indicate that the information quality in the grid is conditional to the number of connections per neuron *𝒟* and that, for a fixed *𝒟*, increasing the absolute number of neurons in a graph is detrimental to information quality in that graph. We introduce now another variable that is herein defined as

(8) $$\begin{array}{c} r_{cm}=\frac{\sum_{i=1}^{N}I_{i}r_{i}}{\sum_{i=1}^{N}I_{i}}, \end{array}$$

where *I*_*i*_ is the information transmitted at a generic node *i* at a distance *r*_*i*_ from the center. Thus, *r*_cm_ indicates the position of the center of mass of information in a network. The larger the *r*_cm_, the further a message travels through the network.  Figure 9 reports *r*_cm_ as a function of *𝒩*. In line with the presented results for *ρ*_*I*_, *r*_cm_ increases for increasing *𝒩* in the low number of nodes range; it reaches an absolute maximum at *𝒩* = 150, and then it decreases. Similarly to *ρ*_*I*_ and depending on the effective links among neurons in a grid, there exists an optimal value of *𝒩* for which information in the domain travels the maximum distance.

### 3.3. The Information Transported over the Grid: Effect of the Latency Time *τ*_*r*_ and Resonance in the Grid

**Figure 10:**
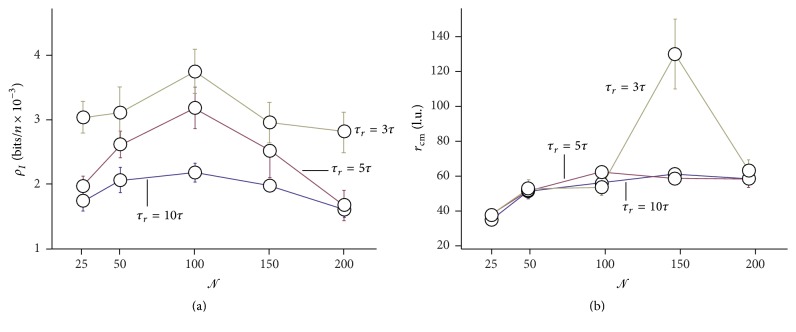
Density of information in a grid of neurons as a function of the number of neurons in the grid, for different refractory times: the smaller the timing of spiking neurons, the larger the information neurons can convey in a grid (a). Position of the center of mass of information in a grid as a function of the number of neurons in the grid, for different refractory times: a combination of network topology and physical characteristics of individual neurons may exist, for which the efficiency of transport is giantly enhanced in a resonance effect (b).

**Figure 11:**
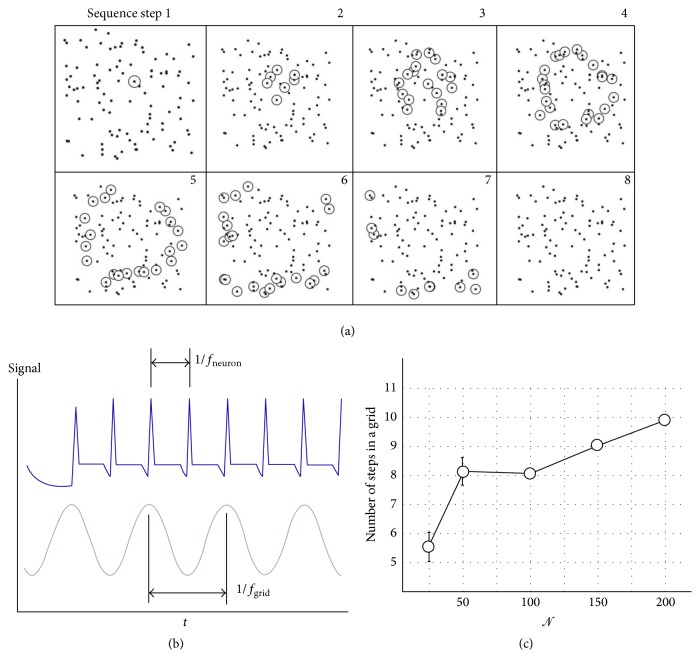
The information that travels through the grid occupies successive sites of the grid in a discrete sequence of steps (a). The timing between successive steps yields the frequency of the grid in contrast to the spiking frequency of individual neurons (b). If the number of steps in a grid assumes an integer value, it implies that the frequency of the neuron is an integer multiple of the frequency of the grid. This generates cumulative amplification of the signal transmitted over the grid.

Here, we present results of simulations in which the refractory time *τ*_*r*_ is varied from 10*τ* down to 3*τ*. The remaining parameters are maintained from the precedent simulations.  Figure 10(a) reports the information density *ρ*_*I*_ as a function of the number of neurons in the grid *𝒩* for different *τ*_*r*_. One may observe that *ρ*_*I*_ increases for decreasing *τ*_*r*_ and this is easily explained considering that a smaller refractory or latency time would translate in a faster firing rate in an individual neuron. In the high *τ*_*r*_ range, that is, moving from *τ*_*r*_ = 10*τ* to *τ*_*r*_ = 5*τ*, a variation in *τ*_*r*_ has the effect to amplify the signal, and the amplification is proportional to the information density at the initial state. For *𝒩* = 100, this scale effect is maximum, with *ρ*_*I*_^*τ*_*r*_=10*τ*^ ~ 0.02 bits/neuron and *ρ*_*I*_^*τ*_*r*_=5*τ*^ ~ 0.03 bits/neuron, and thus the amplification factor is *ξ*_100_ = *ρ*_*I*_^*τ*_*r*_=5*τ*^/*ρ*_*I*_^*τ*_*r*_=10*τ*^ ~ 3/2. Differently, for *𝒩* = 25 and *𝒩* = 200 we register limited and vanishingly small amplification factors *ξ*_25_=*ξ*_200_ ~ 0. In the low *τ*_*r*_ range, that is, moving from *τ*_*r*_ = 5*τ* to *τ*_*r*_ = 3*τ*, decreasing *τ*_*r*_ would not further enhance the* maximum* information density, and this may be ascribed to a* saturation* effect in the grid. Instead, information is globally augmented; that is, information increment is more uniform in the spectrum of *𝒩*, with smaller variations along *𝒩*. Similarly to *ρ*_*I*_, the position of the center of mass of information *r*_cm_ is displayed in Figure 10(b) as a function of *𝒩* for different *τ*_*r*_ = (3,5, 10)*τ*. The diagram of *r*_cm_ may convey even more information on the behavior of the system than *ρ*_*I*_. Notice that while the increment in *r*_cm_ moving from *τ*_*r*_ = 10*τ* to *τ*_*r*_ = 5*τ* is moderate, when the depression time in the neuron is adjusted as *τ*_*r*_ = 3*τ*, we observe an anomalous enhancement of the radius *r*_cm_ at a specific *𝒩* = 150. This* giant* increment may depend on the topology of the network, similarly in concept to the resonance in a mechanical system, and a tentative explanation of a similar effect is provided below. Consider the scheme in Figure 11, individual neurons will emit signals where the time distance between a couple of those signals is the latency or resting time *τ*_*r*_. Thus, the firing rate of individual neurons will be

(9) $$\begin{array}{c} f^{neuron}=\frac{1}{\tau_{r}}=\frac{1}{\chi \tau }, \end{array}$$

where *χ* ∈ {3,5, 10} is an integer. In contrast, the firing frequency of the entire grid will be the reciprocal of the time *τ* ^network^ that the signal takes to travel over the entire network. If we call *s* the number of steps in the discrete sequence that yields a signal from the center of the grid to the periphery, we have

(10) τnetwork=sτ,fnetwork=1sτ

and *s* is a characteristic of the grid. Compare now (9) and (10), you shall find that *f* ^neuron^ = (*s*/*χ*)*f* ^network^; that is, the frequency of the neuron is *s*/*χ* times the frequency of the network. Consider now the inset in Figure 11(c), where the diagram reports the number of steps in a grid *s* as a function of *𝒩* for the considered case *τ*_*r*_ = 3*τ*. You can notice that *s* varies with *𝒩* to a large extent; however, for *𝒩* = 150 it takes the integer value *s* = 9; thus, the frequency of the neuron is an integer multiple *s*/*χ* = 3 of the frequency of the grid. This may generate a cumulative amplification of the signal transmitted over the grid in what resembles a domino effect or chain reaction and may explain the huge distance at which information is transmitted for a similar configuration.

## 4. Discussion and Conclusions

The presented results indicate that in a network of nerve cells the information transmitted over the network depends on the absolute number of cells in the grid and, for intermediate values of neural density, it reaches a maximum. The information is herein represented as the total information (information quantity), that is, the information integrated over all the nodes in the grid, and the density of information, that is, the total information divided per the number of nodes in a network. Moreover, we provide an estimate of the position of the center of mass of information in the net, that is, the distance over which it is transported and the larger the distance the larger the efficiency of the grid (quality of information).

Using mathematical modelling and computer simulations (in which an integrate-and-fire model is coupled to a discrete Shannon's entropy based description of information in bits), we found that the quantity, density, and quality of information depend on the cooperation of neurons in a grid and on the topology of the network. While the information quantity increases as a monotonic function of the number of cells *𝒩* in the domain, the information density and quality have a nonlinear behavior and an optimal value of *𝒩* exists for which they exhibit a maximum. Simple addition of nodes in a network of nerve cells does not enhance the quality of information in that network. Moreover, we found that increasing the firing rate of individual neurons (i.e., reducing the postsynaptic depression time *τ*_*r*_) has the consequent effect of increasing the indexes of information globally in a network. Perhaps more importantly, we found that certain configurations of neurons in a grid may exist for which information in the grid is giantly increased. This is similar in concept to a resonance effect in a physical system in which, when the physical characteristics of the system and the frequency of a field are in specific ratios, the amplitude of the field is amplified.

These findings are in qualitative agreement with other described experiments. Writing on Plos One, Biffi and colleagues [29] demonstrated planar cultures from dissociated primary central neurons using multielectrodes arrays (MEAs). In experiments in which the neural cell density in the culture was varied, they observed a moderate spontaneous cell activity for sparse (900 cells/mm^2^) and dense (3600 cells/mm^2^) cell cultures, differently from medium populated cultures (1800 cells/mm^2^) in which they registered elevated electrophysiological activity in terms of number of active channels, mean frequency, and bursting rate in a network. Commenting on these results, the authors recognized a discrepancy with other reported experiments (to cite a few, the works of Wagenaar et al. [30] and Cohen et al. [31]), in which the spiking frequency decreases moving from low to high cell densities; and vice versa the synchronization among spikes increases, and ascribed a similar discrepancy to coincidental effects, including deviations between nominal and actual seeding densities across different experiments, different culture feeding timings, and different experimental techniques and procedures. However, in an* information theory* interpretation of these data, we propose a diverse explanation of this apparent divergence. Considering that information depends on both the firing rate of signals and the synchronal combination of these signals in a network (and thus network topology cannot be disregarded), the findings of Wagenaar, Cohen, and Biffi and colleagues, rather than contradictory, may in reality support the same notion that, for sufficiently large cell densities, any further increment of neural cells in a network would hamper the transmission of information in that network. Our model is predictive in nature and may recapitulate and explain this sequence of diverse observations.

Our results deserve to be discussed even further. In the simulations, neurons in a plane are connected through a Delaunay triangulation of not-intersecting edges, which guarantees that the number of neurites per neuron is approximately constant and lower than 6 for all the considered configurations (this is described in Section 2 and throughout the paper). Consider now the work of Cullen and colleagues. In [32], they demonstrate that at the early time of synapse formation in planar neural cultures (i.e., for a number of days in vitro DIV from incubation smaller or equal to 7) the mean number of synapses per neuron does not vary and is 5 ± 1 regardless of the cell density in the culture. Thus, our scheme (and noticeably a Delaunay triangulation) may reproduce the transient behavior of nonmature nerve cells at the initial time of network development. Assuming with Gentile [5] that neural cells fate is driven by an information criterion (that would accompany and perhaps conform to an energy and biology criterion), whereby cells on a substrate form patterns that maximize information through those patterns, the presented model and results would explicate the mechanisms of cell adhesion and migration in the early neural cells network.

Consider a certain number of nerve cells seeded on a planar flat surface. Those cells shall be uniformly distributed and thus cell-cell distance would depend on the number of cells and cell density in the culture. If cell density is sufficiently large, internodal distance is small and neurons will develop connecting synapses. Under these conditions, our model indicates that any increase in cell density would adverse information. Thus, any increase in cell density would be prevented and cells would not proliferate nor migrate on the surface. This prediction confirms a number of experiments [5, 33–35] in which it is observed that nerve cells adhesion over planar flat surfaces would not progress or would minimally progress with time after an initial assessment. After synapses formation and neuron-neuron engagement is complete, information transmission in the system is augmented on multiplying the number of synapses among neurons, as observed, for example, in [32]. The presented model and its more sophisticated evolution that will be developed over time may represent a new tool for engineers and neuroscientists for the rational design of scaffolds for applications in regenerative medicine, tissue engineering, and personalized lab-on-a-chips.

## Symbols

*ϕ*: Membrane potential
*C*_*m*_: Capacitance of the membrane
*g*_*l*_: Conductance of the membrane
*R* = 1/*g*_*l*_: Resistance of the membrane
*τ* = *RC*_*m*_: Time constant
*φ*: Resting potential
*θ*: Threshold potential
*I*_stim_(*t*): Stimulating current, which is a function of time
AP: Action potential
*𝒩*: Maximum number of neurons in the grid
*l*: Length of the grid
*𝒫*: Set of neurons in the grid
*P*_*i*_: *i*th element in *𝒫*
*B*_*i*_: Subset of *𝒫* connected to *P*_*i*_
*υ*: Number of edges of the Delaunay triangulation of *𝒫*
*𝒟*: Degree of the graph
*A*: Adjacency matrix
*d*_*ij*_: Distance between *P*_*i*_ and *P*_*j*_
*δ*_co_: Cut-off distance
*δ*_*n*_: Intermodal distance
*J*: Current pulse
*t*_*i*_^*k*^: Timing of individual pulses
rel: Number of neurotransmitter release events
*δ*: Dirac delta function
*H*: Heaviside function
*ζ*: Attenuation
*τ*_*r*_: Refractory or resting time
Δ*τ*: Time bin
*S*^*o*^: Total entropy
*S*^*N*^: Noise entropy
*T*: Temporal window in which the signal is transmitted
*w*: Word, sequence of binary events in *T*
*p*(*w*): Probability of a word
*I*: Information
*r*: Radial distance from the central node in a network
*I*_*r*_: Information transmitted at a generic distance *r*
*I*_tot_: Total information integrated over the entire grid
*ρ*_*I*_: Information density
*r*_cm_: Center of mass of information
*ξ*: Amplification factor
*f*: Frequency
*s*: Number of steps in a grid.
